# Tubular cell damage may be the earliest sign of renal extrahepatic manifestation caused by Hepatitis C

**DOI:** 10.1371/journal.pone.0251392

**Published:** 2021-05-07

**Authors:** Kati Kaartinen, Sauli Vuoti, Eero Honkanen, Eliisa Löyttyniemi, Ravinder Singh, Martti Färkkilä

**Affiliations:** 1 Helsinki University Hospital, Helsinki, Finland; 2 Department of Medicinal Chemistry, University of Jyväskylä, Jyväskylä, Finland; 3 Department of Biostatistics, University of Turku, Turku, Finland; 4 MSD Norway, Drammen, Norway; National Taiwan University Hospital, TAIWAN

## Abstract

Chronic kidney disease (CKD) is one of the most well-known extrahepatic manifestations caused by hepatitis C infection (HCV). CKD is typically discovered at a late stage. HCV-nephropathy may show different histopathologic patterns, as both glomerular and tubulointerstitial damage have been described. Identification of patients with early renal manifestations would be beneficial to provide treatment and avoid progression to CKD. The observational prospective single-center HCVKID study assessed the prevalence of early renal manifestations in patients with chronic HCV and compared these patients with HCV-negative healthy controls cross-sectionally. HCV-positive patients with and without renal manifestations were also compared to define biomarkers suitable for identifying early manifestations in standard clinical practice. Tubular proteinuria as judged by urine α 1-microglobulin was the most common early renal manifestation found in 11% in HCV-positive patients, followed by hematuria in 8%. Kidney filtration was statistically significantly lower among HCV-positive patients with renal manifestation according to any calculation method. There were no significant differences in duration of infection or stage of liver fibrosis between patients with or without renal manifestations. Tubular cell damage may be the earliest sign of renal dysfunction caused by HCV. Complement activation also correlates with the dysfunction, indicating of contribution to HCV-induced renal manifestations even in their early phase.

## Introduction

Hepatitis C [HCV] infection is known to cause kidney manifestations, which are typically discovered at a late stage when chronic kidney disease (CKD) has already emerged [1–3]. HCV is both a consequence and cause of renal impairment: first, dialysis patients have an increased infection risk related to medical procedures and, second, HCV causes pathological changes to the kidneys [1, 4, 5]. Membranoproliferative glomerulonephritis [MPGN] either with or without linkage to mixed cryoglobulinemia has been reported as the most common renal manifestation. Overall, there is an over two-fold increase in the risk of end-stage renal disease [ESRD] for all HCV-infected patients, and nearly seven times higher risk in those HCV-infected patients aged 50–59 compared to non-infected patients [6]. Recent meta-analyses have shown that patients with HCV were at a 23–54% greater risk of presenting with CKD compared to non-infected patients, with considerable differences in country-specific figures [7, 8]. Annual eGFR decline was 0.58 ml/min higher for HCV-positive compared to HCV-negative CKD 3–5 patients [9]. CKD was four times more common among HCV patients compared to the general population in a recent registry study, and the mortality of untreated HCV patients with CKD was two times higher compared to those receiving treatment and generated high treatment costs [10]. However, reports of kidney manifestations before the onset of CKD among HCV-positive are rare.

The risk for proteinuria [either based on an assessment of a urine protein dipstick test or urine [micro] albuminuria/creatinine ratio] increases by 29% with a hepatitis C infection according to a meta-analysis of 10 studies describing CKD patients [8]. The prevalence of kidney involvements among HCV-positive persons examined using a wide array of diagnostic methods was as high as 40% in a medium-size cohort [11]. The most frequent risk factors for kidney involvement have been advanced age, extensive liver fibrosis and a chronic HCV infection [12, 13].

HCV-related nephropathy may appear at any time during the natural history of an HCV infection [14]. While it may show different histopathologic patterns, as both glomerular and tubulointerstitial damage have been described, the underlying mechanisms remain unclear [14–18]. Subclinical glomerulopathy is frequently present during the course of the HCV infection [18], but renal disease associated with HCV is often left unnoticed [19]. Kidney injury may result from immune-mediated tissue damage or from direct effects of HCV, and other yet unknown mechanisms may also be involved [14].

The aim of this observational single-center HCVKID study was to evaluate the prevalence of early renal manifestations among patients with chronic HCV yet without documented CKD, and compare the patient population with HCV-negative controls cross-sectionally. The study also aimed to find means to identify patients with early kidney manifestations and to improve the understanding of the mechanisms leading to the onset of CKD. This would provide the opportunity to identify and treat the patients before the onset of CKD and occurrence of irreversible kidney damage, and reduce the global burden of CKD among HCV-positive.

## Materials and methods

### Patients

**Fig 1:**
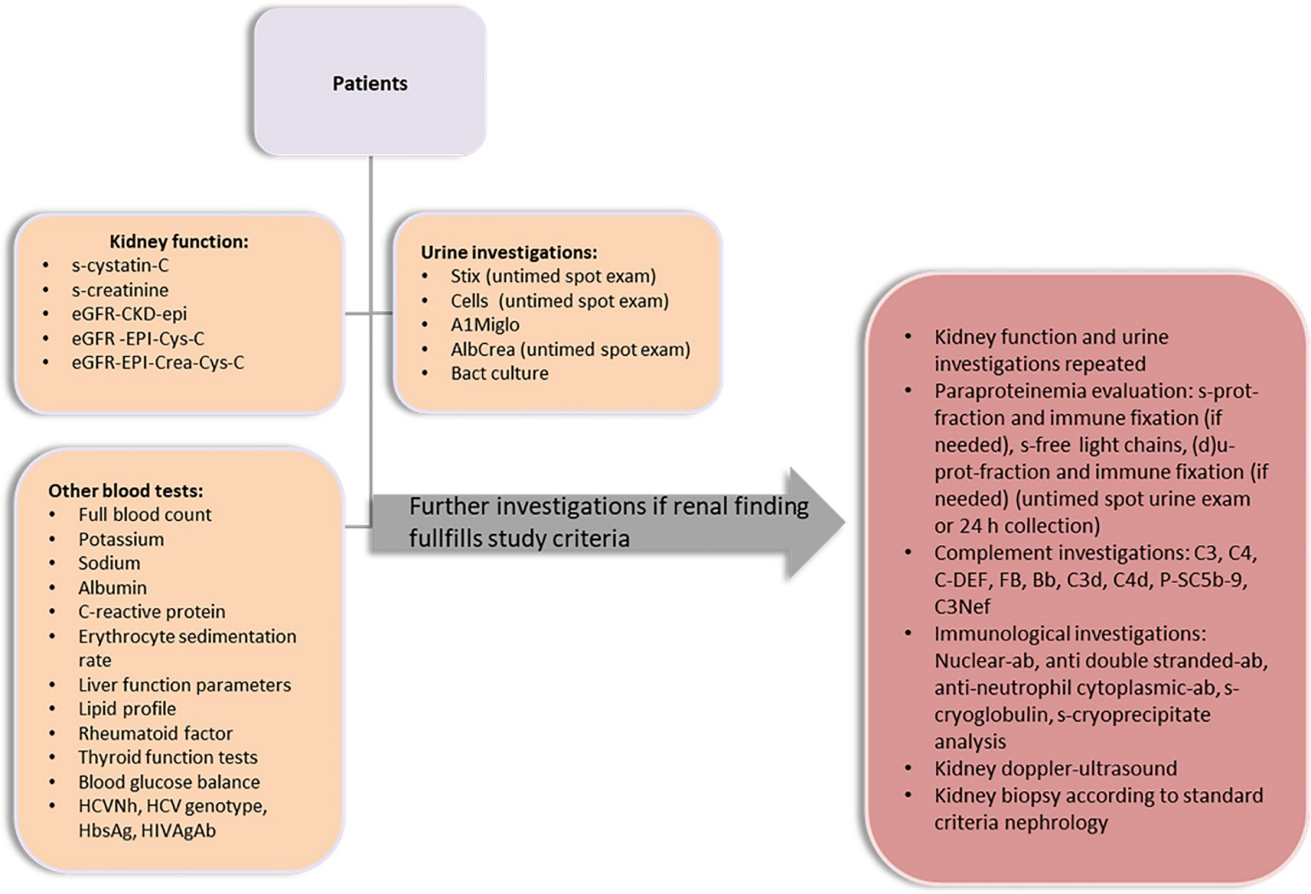
Study flow chart.

A total of 211 HCV-positive patients were consecutively recruited by the Department of Gastroenterology at the Helsinki University Central Hospital, Finland (see Fig 1) during 2017–2018. Blood and urine samples are collected from all patients as a standard clinical procedure. The patients had been referred to the assessment of treatment by primary healthcare providers after the patients’ HCV-positive status had been confirmed (HCVNh positive). No administration of any therapeutic or prophylactic agents was required by the protocol. HCV-negative controls were recruited among individuals seen for routine health examinations by an occupational healthcare provider during roughly the same 24-month period. As blood and urine samples were required for the purposes of the present study, the patients who had scheduled a blood test as part of their occupational healthcare appointment were identified as potential HCV-negative, healthy controls.

Inclusion criteria were:

1. All: Adult male or female patients (≥ 18 years old)
2. HCV-positive patients: HCV status confirmed by entries in medical records on HCV-RNA and a referral for assessment at the Helsinki University Hospital’s Department of Gastroenterology
3. HCV-negative controls: examined in a routine health appointment by an occupational healthcare provider
4. All: Providing written informed consent

Exclusion criteria were:

1. All: HIV-positive patients
2. All: prior history of renal disease
3. All: Hepatitis B positive (HbsAg) patients
4. HCV-negative controls: Diabetes, hypertension requiring medication, other underlying diseases that influence kidney function (e.g. chronic rheumatoid arthritis)
5. HCV-positive patients: Other underlying diseases affecting kidneys unless associated with an extrahepatic manifestation caused by HCV. Each patient with such other conditions was separately assessed by the researchers to determine whether the condition (e.g. diabetes) had emerged before the HCV infection. All patients with underlying diseases affecting kidney function diagnosed before the chronic HCV were excluded.

The complete study population comprised 492 healthy controls and 211 HCV-RNA positive patients. In total, 13 patients withdrew their consent and one patient was excluded due to a previously diagnosed biopsy proven renal disease (mesangioproliferative glomerulonephritis). The study was conducted in accordance with the ethical principles that originating from the Declaration of Helsinki. Informed consent was obtained when patients first visited the clinic or had their routine health appointment with the occupational healthcare provider. All patients and controls gave their signed informed consent. The study protocol was approved by the Helsinki University Hospital (HUS) Ethical Committee, decision number HUS/1264/2016.

#### Laboratory methods

In this study, the definition of renal manifestation was based on the evaluation of renal function and urine analysis according to international standards. An HCV-positive patient was considered to have a renal manifestation if his or her s-creatinine was above the upper normal limit (male > 100 μmol/l, female > 90 μmol/l) or if his or her estimated glomerular filtration rate (eGFR) calculated according to the Chronic Kidney Disease Epidemiology Collaboration (CKD-EPI) equation was below the normal limit (< 60 ml/min/ 1.73 m2). Renal manifestation based on urine analyses was defined as either the number of red blood cells above the normal limit in urine microscopy (u–erythrocytes ≥ 20 x 10(E6)/l), tubular proteinuria above the normal limit (u–α 1-microglobulin, A1M, ≥ 12 mg/l) or glomerular proteinuria above the normal limit (u–albumin/creatinine ratio, albcrea, in men ≥ 2.5 mg/mmol, in women ≥ 3.5 mg/mmol) in a spot urine test. Those with any renal manifestation were referred to the Department of Nephrology for further standard examinations.

The HCV-negative controls were considered to have renal impairment if their s-creatinine was above the upper normal limit (male > 100 μmol/l, female > 90 μmol/l) or if their eGFR calculated according to the Chronic Kidney Disease Epidemiology Collaboration (CKD-EPI) equation fell below the normal limit (< 60 ml/min/ 1.73 m2). Renal manifestation based on urine analyses was defined as either the number of red blood cells exceeding the normal limit in urine microscopy (u–erythrocytes ≥ 20 x 10(E6)/l) or a spot urine dipstick test revealing any amount of proteinuria (trace, +, ++, +++). Those with renal manifestations were referred to their occupational physicians for further examination.

Alcohol consumption was assessed using a standard questionnaire (AUDIT) among the HCV-positive patients. The estimated time and route of HCV transmission was also evaluated based on patient interviews.

All blood samples were taken after receiving informed consent from the research subjects and before their appointments at the Department of Gastroenterology or Nephrology. Urine samples were collected as spot urine samples and an additional 24-h urine collection was conducted before the appointments at the Nephrology department. Laboratory variables were analyzed at the accredited Helsinki University Hospital laboratory (HUSLAB) using standard chemical methods.

Kidney function was measured and estimated using five different approaches: serum creatinine, serum cystatin-C and eGFRs utilizing three different formulas. Estimated GFRs were calculated according to the Chronic Kidney Disease Epidemiology Collaboration equation (CKD-EPI), CKD-EPI Cystatin C equation (eGFR-EPI-cys-C) and CKD-EPI creatinine-cystatin C equation (eGFR-EPI-crea-cys-C) (S-creatinine) was determined with the enzymatic method of Abbott Laboratories. Upper normal values for serum creatinine were 100 μmol/l in males and 90 μmol/l in females. S-cystatin-C was assessed by multigent cystatin C 1P93-30 method by Abbott Laboratories and was considered normal when < 1 mg/l (under 50 years of age) or < 1.2 mg/l (> 50 years of age). The cut-off for lower normal value was 60 ml/min/ 1.73 m2 for all eGFRs.

Urine A1M as a marker of tubular proteinuria was measured from a spot urine sample taken in the morning using the turbidimetry method of Abbott Laboratories. Values <12 mg/l were considered normal. Urine albcrea was measured as a marker for glomerular proteinuria based on a spot urine sample taken in the morning and involved analyzing albumin with the albumin BCP method and creatinine with the enzymatic method by Abbott Laboratories. Values < 2.5 mg/mmol for males and < 3.5 mg/mmol for females were considered normal. A 24-h urine collection was considered normal if the amount of proteinuria was < 100 mg/24-h. Urine hemoglobin was tested using both dipstick test and automated phase contrast microscopy analysis. The latter was used in addressing whether the subject had hematuria. The hospital laboratory changed into using an automated microscopy analysis by the end of April 2019. The upper normal value of u–erythrocytes was 20 x 10 (E6)/l before this and 10 x 10 (E6)/l after the change.

Complement analyses were performed using the Wieslab complement system screen method (activity percentages of serum classical, alternative and lectin pathways, S -CH100Cl, S -CH100Al, S -CH100L, respectively) and nephelometry (serum complement 3 and 4, S -C3, S -C4, respectively) by Abbott Laboratories. C3Nephritic factor (C3Nef) was analyzed by in-house immunofixation electrophoresis, which involved examining the ability of the patient’s serum to activate an alternative pathway of complement in normal serum in the presence of magnesium ethylene glycol tetra-acetic acid (MgEGTA). Complement activation products were analyzed using several methods, radial immunodiffusion (complement 4 activation product, C4d and factor B, Facb), rocket immunoelectrophoresis (complement 3 activation product, C3d) and enzyme immunological methods (factor B activation product, FacBb, membrane attack complex, SC5b-9) in the accredited laboratory of Turku University Hospital.

Serum free light chains were analyzed using immunoturbidimetric assay by the Binding Site, Birmingham, UK. Serum paraproteins were analyzed using capillary electrophoresis by Sebia, Paris, France and urine paraproteins from the 24-h urine sample with agarose gel electrophoresis followed by immunofixation by Sebia, Paris, France.

Aspartate aminotransferase to platelet ratio index (APRI) was used to predict significant liver fibrosis (35). Values < 0.5 refer to significant fibrosis to be unlikely whereas values > 1.5 refer to likely significant fibrosis. Liver fibrosis was also assessed by transient elastography (FibroScan®, Echosens, Paris, France) after a 4-hour fasting. Liver biopsy was available for 10 patients.

#### Statistical analyses

In the original study protocol, the sample size estimations were based on the prevalence of any type of renal involvement in patients with HCV compared to healthy controls. Due to the lack of literature regarding renal deterioration as a general term, we conducted the sample size calculation using prevalence of late stage 4–5 chronic kidney disease (CKD) and end-stage renal disease (ESRD) as possible renal diseases observed in this study. The estimated sample size per study group ranged between 250 to 500 subjects. However, after recruitment of 104 patients, it was noted that the prevalence was 20% among the patients, and 0% in the healthy controls according to the protocol-defined limits. This led to the possibility to decrease the amount of recruited patients, thus facilitating the final analysis and reporting the study results. Therefore, new sample size estimations were performed.

In the updated protocol, the intention was to collect approximately 200 patients and 400 healthy controls. According to these updated calculations, a sample size of 140 patients per study group with an assumed prevalence of any renal manifestation being at least 15% among the HCV-positive patients and prevalence of 5% at most in the healthy controls would already lead to statistical power of at least 80% (significance level of 5%, two-tailed) for detecting statistically significant differences.

Continuous variables were reported as the number of patients whose data were summarized (n), mean, standard deviation (SD), and minimum and maximum. All categorical variables were reported using frequency counts and percentages by category.

Comparisons of baseline characteristics were made between HCV-positive patients with renal manifestation and the HCV-positive patients without renal manifestation, and also between all patients against the HCV- negative controls. Fisher’s exact test was performed for categorical variables, and Student’s t-test if the variable followed normal distribution, Wilcoxon rank sum test was otherwise used. For some variables, logarithmic transformation was applied to achieve normal distribution.

In any of the statistical comparisons, two-sided tests were used at α = 0.05 significance level. In general, missing data were not imputed and the data were analyzed as they had been recorded, unless otherwise specified in the SAP. All statistical analyses and generation of all tables, listings and figures were performed using the SAS® (SAS Institute, North Carolina, USA) software, version 9.4.

## Results and discussion

### Demographic characteristics between healthy controls and HCV patients

Complete data were available on 211 patients. The baseline characteristics of the HCV-negative controls compared to the HCV-positive patients are shown in Table 1. The healthy controls had a lower rate of diabetes and hypertension, were more often non-smokers and had a lower BMI compared to HCV-positive subjects. The protein dipstick test was positive in 0.2% (1/492) among healthy controls (p = 0.03 compared to HCV-positive) and hematuria was found in 5% (23/492) (p = 0.42 compared to the HCV-positive patients) (Table 1). However, in gender-specific analyses, HCV-positive females had significantly more hematuria compared to healthy controls (13% vs. 6%, p = 0.04). In males, the corresponding figures did not show a significant difference even if slightly higher in HCV-positive (6% vs. 4%, p = 0.31).

**Table 1:** Baseline characteristics of HCV-positive patients and HCV-negative controls. Values are expressed as means (range or standard deviation, SD, in parentheses) or number of patients (percentages in parentheses). Reference values and units are given where appropriate.

Baseline variable	HCV- positive n = 211	Controls n = 492	p-value
Age (years), (range)**	43.6 (20–77)	35.9 (20–65)	<0.0001
Race			
Caucasian *n* (%)	199 (94)	492 (100)	
non-Caucasian *n* (%)	12 (6)	0 (0)	
Male gender *n* (%)	114(54)	240 (49)	0.22
Diabetes *n* (%)	12 (6)	1 (0)	
Hypertension *n (*%)	29 (14)	3 (1)	
Rheumatic disease *n* (%)	7 (3)	9 (2)	0.27
Smoking			
No (%)	33 (16)	358 (73)	<0.0001
Yes (%)	124 (59)	48 (10)	
Former (%)	54 (26)	86 (17)	
BMI (kg/m^2,^)*	26.0 (4.5)	25.2 (4.2)	0.03
eGFR CKD-EPI (≥ 60 ml/min/1.73m^2^)*	106.0 (14.1)	107.9 (14.6)	0.12
S-creatinine (≤ 100 μmol/l male, ≤ 90 μmol/l female)*	66.6 (12.6)	69.9 (13.3)	0.003
Higher than normal *n (%)*	6 (3)	13 (3)	0.80
Urine dipstick positive for proteinuria *n* (%)	4 (2)	1 (0)	0.03
Urine microscopy positive for hematuria *n (*%)	16 (8)	23 (5)	0.10

*mean (SD)

** mean (range). BMI = body mass index, eGFR CKD-EPI = estimated glomerular filtration rate, calculated according to the Chronic Kidney Disease Epidemiology Collaboration (CKD-EPI) equation.

### Clinical characteristics in HCV-positive patients with and without renal manifestation

Proteinuria was the most common urine manifestation among the HCV-positive patients. The protein dipstick test was positive in 2% (4/211), above normal tubular proteinuria, A1M, in 11% (22/210) and above normal albuminuria, albcrea, in 3% (6/210) of the patients. Hematuria was found in 8% (16/211) of the patients. Serum cystatin-C was elevated in 35% (73/210), but s-creatinine only in 2.9% (6/210) of all HCV-positive patients. S-creatinine was elevated in 2.6% (13/492) of the control group (p = 0.11 compared to the HCV-positive patients). EGFR according to the Chronic Kidney Disease Epidemiology Collaboration (CKD-EPI) equation was not significantly different between healthy controls and HCV-positive patients (Table 1). The proportion of different renal manifestations has been shown in S1 Fig.

The characteristics of the HCV-positive patients with and without renal manifestation were similar. There were no significant differences between the groups in the estimated time from HCV transmission, route of infection, viral load and genotype, use of alcohol and stage of liver fibrosis (Table 2). The most common genotype was 3 followed by 1 in both groups, corresponding to the national epidemiology rates. None of the patients had genotypes 5 or 6 or a mixed genotype, which is known to be rare in Finland. Most of the patients (62%) had only a minor liver fibrosis (stage F0-F1) and only 15% had a stage 4 (F4) liver fibrosis. In most of the patients, the main source of the HCV infection was related to previous intravenous drug abuse. A significantly higher number of the patients without renal manifestation were receiving opioid substitution therapy (OST). Aspartate aminotransferase to platelet ratio (APRI) index and liver variables were similar among both HCV-positive groups.

**Table 2:** Baseline characteristics of the HCV-positive patients with and without renal manifestation. Values are expressed as means (range or standard deviation, SD, in parenthesis) or number of patients (percentages in parenthesis). Reference values and units are given where appropriate.

Baseline variable	HCV-positive with renal manifestation n = 41	HCV-positive with no renal manifestation n = 170	p-value
Age (years)**	45.4 (20–70)	43.2 (20–77)	0.31
Race			
Caucasian *n* (%)	38 (93)	168 (95)	0.71
non-Caucasian *n* (%)	3 (7)	8 (5)	
Male gender *n* (%)	20 (49)	94 (55)	0.49
Diabetes *n* (%)	3 (7)	9 (5)	0.71
Hypertension *n (*%)	8 (20)	21 (12)	0.31
Rheumatic disease *n* (%)	0 (0)	7 (4)	0.35
Smoking			
No (%)	4 (10)	29 (17)	0.09
Yes (%)	21 (51)	103 (61)	
Former (%)	16 (39)	38 (22)	
BMI (kg/m^2,^)*	26.3 (4.6)	26.0 (4.6)	0.64
APRI (> 1.0)**	1.4 (0.2–14.7)	1.1 (0.2–7.4)	0.19
Genotype *n* (%)			
1	18 (44)	71 (42)	0.06
2	1 (4)	22 (13)	
3	21 (51)	77 (45)	
4	1 (2)	0 (0)	
Fibrosis stage *n (*%)			
F0-F1	24 (60)	107 (65)	0.54
F2	5 (13)	28 (17)	
F3	3 (8)	7 (4)	
F4	8 (20)	23 (14)	
Route of infection *n* (%)			
former drug use	29 (71)	127 (75)	0.38
sex	4 (10)	10 (6)	
blood	0 (0)	9 (5)	
unknown	6 (15)	15 (9)	
other	2 (5)	9 (5)	
Estimated time in years for duration of HCV infection**	18.8 (1–42)	20.7 (1–49)	0.39
Use of alcohol n (%)			
Never	19 (46)	66 (39)	0.65
Once monthly or less	10 (24)	49 (29)	
2–4 times monthly	9 (22)	30 (18)	
2–3 times weekly	2 (5)	20 (12)	
4 times or more weekly	1 (2)	5 (3)	
OST therapy	2 (5)	42 (28)	0.005

*mean (SD)

** mean (range). BMI = body mass index, APRI = aspartate aminotransferase to platelet ratio index, OST = opioid substitution therapy.

Baseline blood values are presented in S1 Table. There were no significant differences between the groups. As the only exception, hemoglobin was significantly higher for the HCV-positive females with renal manifestation. None of the patients had cryoglobulinemia based on serum analyses nor positive results in anti-neutrophil cytoplasmic or anti-double stranded antibodies.

Table 3 shows the renal variables in detail. All findings among patients with renal manifestation were significantly different compared to no renal manifestation–group. A chart describing detailed parallel findings and individual level renal manifestations is presented as S2 Table. Proteinuria and hematuria were usually separate findings, with only 10% (4/41) of the patients having concurrent hematuria and proteinuria (S2 Table). Three patients with renal manifestation had elevated creatinine as the only finding without hematuria or proteinuria (S2 Table). Interestingly, the number of patients with abnormal renal function was heavily dependent on the method of evaluation ranging from 0.5% in the CKD-EPI equation to 2% in the eGFR-EPI-crea-cys-C equation and reaching 17% in the eGFR-EPI-cys-C equation. Overall, solely cystatin-C based methods indicated a higher number of patients with abnormal renal function compared to solely creatinine-based methods (35 vs. 1). S-creatinine and equation based on both s-cystatin-C and s-creatinine (eGFR-EPI-crea-cys-C) indicated similar results, 3% and 2%, respectively. The difference between the manifestation vs. no manifestation was significantly different based on any of the filtration methods.

**Table 3:** Baseline values of renal laboratory variables in HCV-positive patients with renal manifestation compared to no renal manifestation. Reference values and units are given where appropriate. Values are expressed as means (range or standard deviation, SD, in parenthesis). No patient had eGFR less than 45 ml/min/1.73m^2^.

Baseline variable (reference values, units)	HCV positive with renal manifestation n = 41	HCV positive with no renal manifestation n = 157–169	p-value
CKD-EPI (≥ 60 ml/min/1.73m^2^)*	98.8 (18.9)	107.8 (12.0)	0.0002
eGFR-EPI-cys-C (≥ 60 ml/min/1.73m^2^)*	71.2 (22.8)	83.5 (19.6)	0.0007
eGFR-EPI-crea-cys-C (≥ 60 ml/min/1.73m^2^)*	82.2 (19.7)	94.0 (15.3)	<0.0001
S-creatinine (≤ 100 μmol/l male, ≤ 90 μmol/l female)*	71.8 (18.8)	62.3 (10.9)	0.003
S-cystatin-C (< 1.0 mg/l ≤ 50 years, <1.2 mg/l > 50 years)**	1.20 (0.76–3.86)	1.00 (0.61–1.55)	0.0001
Urine microscopy positive for hematuria *n (*%)	17 (43)	1 (1) ***	<0.0001
Urine A1M (< 12 mg/l)**	14.5 (5.0–70.2)	6.1 (1.6–11.8)	<0.0001
Urine albcrea (< 2.5 M, < 3.5 F mg/mmol)**	7.3 (0.3–167.8)	0.9 (0.3–3.4)	<0.0001
CKD G1 (eGFR ≥ 90 ml/min/1.73m^2^) n (%)	30/41 (74%)	1/168 (0.6%)	0.0006
CKD G2 (eGFR 60–89 ml/min/1.73m^2^) n (%)	10/41 (24%)	0/168 (0%)	
CKD G3a (eGFR 45–59 ml/min/1.73m^2^) n (%)	1/41 (2%)	0/168 (0%)	

*mean (SD)

** mean (range)

***. One patient with hematuria was later found in the no renal manifestation group due to the change in reference values (20 →10) in the amount of red blood cells in urine during the late phases of the study. His previous and subsequent urine tests showed no abnormality, therefore analyses were kept unchanged. CKD-EPI = estimated glomerular filtration rate, calculated according to the Chronic Kidney Disease Epidemiology Collaboration (CKD-EPI) equation, eGFR-EPI-cys-C = estimated glomerular filtration rate, calculated using cystatin-C, eGFR-EPI-crea-cys-C = estimated glomerular filtration rate, calculated using creatinine and cystatin-C. The equations are presented in Inker et. al 2012. A1M = urine α 1-microglobulin, albcrea = urine albumin/creatinine ratio.

Urine A1M was not significantly correlated with the variables linked to a chronic HCV infection (genotype, HCV viral load, estimated time of transmission and stage of fibrosis or liver cirrhosis based on data obtained from electronic health records combining clinical data, biopsy and FibroScan). Urine albcrea was significantly correlated with the estimated time of transmission (p = 0.04), diabetes (p = 0.03) and liver cirrhosis based on electronic health records combining clinical data, biopsy and FibroScan results (p = 0.003).

### Monoclonal paraproteins and complement analyses in HCV-positive patients with renal manifestation

An analysis of monoclonal serum and urine paraproteins, and blood complement variables was only performed on the group with renal findings. The results are presented in Table 4. Serum monoclonal protein was found in 2.8% (1/36) (IgM kappa) and in 7.4% (2/27) (kappa light chain) of the patients based on the 24-h urine collection. While the mean ratio of kappa/lambda serum free light chains was within normal limits, the range fell below or exceeded the normal values in 31% of the patients.

**Table 4:** Complement and monoclonal paraprotein variables in HCV-positive patients with renal manifestation. Reference values and units are given where appropriate. Values are expressed as means (range in parenthesis) or number of patients (percentages in parenthesis).

Variable	n = 37
**Complement proteins (reference)**	
S-C3 (0.5–1.5 g/l)	1.14 (0.71–2.14)
Less than normal n (%)	0 (0)
S-C4 (0.12–0.42 g/l)	0.21 (0.04–0.76)
Less than normal n (%)	4 (11)
p-FacB (0.1–0.4 g/l)	0.20 (0.12–0.31)
Less than normal n (%)	0 (0)
**Functional complement analyses**	
S-CH100Al (> 39%)	102.54 (51–173)
Less than normal n (%)	0 (0)
S-CH100Cl (> 74%)	101.27 (0–200)
Less than normal n (%)	7 (19)
S-CH100L (> 10%)	73.7 (0–198)
Less than normal n (%)	7 (19)
**Complement activation products**	
p-C4d (< 7 μg/ml)	2.64 (0.5–8.7)
More than normal n (%)	3 (8)
p-C3d (< 7 U/ml)	5.52 (2.9–10.7)
More than normal n (%)	5 (14)
p-FacBb (< 4 μg/ml)	1.13 (0.1–2.4)
More than normal n (%)	0 (0)
p-SC5b-9 (< 366 ng/ml)	183.11 (42–349)
More than normal n (%)	0 (0)
**Complement autoantibody**	
C3nef (positive) n (%)	4/37 (11%)
**Paraproteins**	
S-IgLcK (6.9–25.6 mg/l)	28.1 (9.6–104.0)
Outside reference range n (%)	13 (36)
S-IgLcL (8.6–26.5 mg/l)	22.4 (9.0–51.9)
Outside reference range n (%)	9 (25)
S-K/L-S-ratio (0.52–1.40)	1.23 (0.40–2.18)
Outside reference range n (%)	11 (31)
Serum paraprotein (no) n (%)	35/36 (97%)
Urine paraprotein (no) n (%)	25/27 (93%)

C3 = complement 3, C4 = complement 4, CH100Al = activity of the alternative pathway of complement, CH100Cl = activity of the classical pathway of complement, CH100L = activity of the lectin pathway of complement, C4d = complement 4 activation product, C3d = complement 3 activation product, FacB = factor B, FacBb = factor B activation product, SC5b-9 = membrane attack complex, C3nef = complement 3 nephritic factor, IgLcK = immunoglobulin light-chain kappa, IgLcL = immunoglobulin light-chain lambda, K/L = kappa/lambda.

None of the patients had a below normal s-C3 value, but 11% had a below normal s-C4 value. C3nef was positive in 10.8% (4/37). The activity of classical and lectin pathways of complement were lower than normal in 19% of the patients (both pathways), and as a sign of complement activation, the activity products of classical and alternative pathways were increased (Table 4). The frequency of complement activation or paraproteinemias were more often found in those with proteinuria at 37% (15/41) compared to in those with hematuria at 17% (7/41) (S2 Table). Urine A1M correlated significantly with complement activation products FacBb and SC5b-9 (p = 0.01 for both comparisons). Urine albcrea correlated significantly with C3 and activation product FacBb (p = 0.02 and p = 0.03, respectively). The results of our study showed that the clinical characteristics of HCV-positive patients and healthy controls were surprisingly similar. HCV-positive patients had statistically significantly lower median s-creatinine, and proteinuria was common among HCV-positive according to detailed proteinuria analytical methods. However, a simple pre-screening proteinuria dipstick test utilized routinely in clinical practice did not correlate with the findings discovered using the detailed methods. This is in line with the conclusion in previous studies where kidney manifestations were typically found at CKD stages 3–5 [1–5] and were not identified with dipstick tests at early stage.

Diabetes and hypertension, which have been previously associated with HCV and CKD, were equally frequent among HCV patients with or without renal manifestation according to our classification. To our surprise, hematuria was as frequent among the healthy control group as in the HCV-positive patients, but a gender-specific analysis showed that HCV-positive females had more hematuria compared to the healthy female control group. However, hematuria in the context of HCV infection can be clinically more significant.

Interestingly, genotype, HCV viral load, estimated time of transmission and stage of fibrosis, which have been previously speculated to be associated with HCV-induced chronic kidney disease [11, 12], were not associated with renal manifestations in our study. This was the case despite the fact that we utilized a wide range of renal tests based on several filtration as well as urine markers, and the estimated time since HCV transmission was generally long.

Our results based on proteinuria profiles indicated tubulointerstitial damage rather than glomerular damage at early stages of renal manifestations. According to clinical classification, 15% of the HCV patients were at CKD stage G1, 5% at CKD stage G2 and only 0.5% at CKD stage 3a. Abnormally high urine A1M was an infrequent finding among those with diabetes (normal A1M in 9/11 vs. higher than normal A1M in 2/11, p 0.055). Also, abnormally high urine A1M was a significantly rare finding among those with hypertension (normal A1M in 22/27 vs. higher than normal A1M in 5/27, p 0.0007). The occurrence of diabetes and hypertension was similar among patients with or without kidney manifestations. While an HCV infection seems to increase the risk of proteinuria independently, estimates are usually based on an evaluation of glomerular proteinuria that rely on either the albumin/creatinine-ratio, urine albumin or dipstick test [3, 8]. The results of an Italian study of 98 cirrhotic patients, whose most prominent renal finding was tubular involvement [11], are also in line with our findings. The presence of HCV RNA by in situ hybridization showed evidence of positive hybridization signals in the tubular epithelial cells and tubulointerstitial blood vessels in patients biopsied due to either severe urine findings or decreased renal function [15]. In their study, the majority had nevertheless glomerulonephritis and none had tubulointerstitial nephritis. Another study investigating HCV RNA and HCV core protein in glomerular and tubular structures showed their different distribution potentially reflecting distinct pathways of HCV-related damage in glomeruli and tubules [16]. The HCV core protein was encountered clearly more frequently in the tubules, while both were encountered in the glomeruli. Again, the population comprised patients who had glomerulonephritides with substantial clinical renal findings necessitating a renal biopsy [17]. As renal biopsy is an invasive procedure, minor urine findings do not justify the procedure. Therefore, it can only be speculated that if an earlier biopsy or an alternative analysis method was possible, it could potentially reveal anomalies mostly located in the tubules. Initial tubular damage may contribute to glomerular changes after an unknown period simultaneously reflecting transition from reversible damage to more severe, and potentially irreversible, damage. HCV has been shown to directly affect tubular barrier function in renal epithelial cells by activating the caspases 3, 8 and 9, which favor the apoptosis cascade in renal proximal tubular epithelia [20]. The prevalence of tubular proteinuria in the general population has been generally less than 1% [21].

Despite the long exposure to an HCV-infection, none of the patients in our study had cryoglobulinemia or MPGN, and the effect of HCV on urine findings was mostly apparent as proteinuria since hematuria was equally rare as among the healthy controls. Nationwide, population-based, Israelian retrospective cohort study of 1.2 million persons aged 16–25 years [60% male] revealed isolated microscopic hematuria in 0.3% of the individuals [22] while isolated hematuria in a Korean population study showed incidence of dipstick hematuria in age group of the 20‘s and 30’s to be as high as 22.6% and 25.8% [23]. In our study 5% of healthy controls and 8% of HCV-positive patients had hematuria, which is much more than in the Israelian study, but clearly less than in the Korean study. Isolated hematuria may represent as the only finding in glomerulonephritides and therefore remains important to screen among HCV patients [18, 24]. As indicated above, membranoproliferative glomerulonephritis [MPGN], either with or without linkage to mixed cryoglobulinemia, has been reported as the most common renal manifestation [4]. However, recent figures point to very small numbers of cases with both MPGN and cryoglobulinemia with a reported incidence of 0.22% in HCV patients [25]. In contrast, in a Japanese autopsy study on 188 consecutive HCV patients, only 45% had normal or nearly normal renal histology, while the rest had various forms of glomerulonephritides. In this study, continuous proteinuria or microhematuria had been reported in 12% of the cases one year prior to the patient’s death; however, the incidence of abnormal urine findings was much lower compared to that of biopsy-proven glomerulonephritides. Information on the estimated time of the HCV transmission or renal function was, however, not provided [26]. In two smaller biopsy studies, the presence of cryoglobulins was found in the majority of HCV-patients with MPGN, strongly indicating an etiological relationship between cryoglobulinemia and MPGN [27, 28]. Furthermore, in a study that involved performing renal biopsies on patients with cirrhosis due to chronic HCV infection at the time of liver transplantation, immune complex-mediated glomerulonephritis was found in the majority of the patients (25/30) [29]. An extensive range of renal diagnoses was also found in a medium size Japanese biopsy study of 68 HCV patients, including various glomerulonephritides in 57% and tubulointerstitial nephritis in only 3% of the patients [30].

Monoclonal gammopathies have also been described as extrahepatic manifestations in HCV [12, 31, 32]. Serum and urine paraproteins were found in 2.8% and 7.4%, respectively, of patients who were classified as having renal manifestations.

Antigen-antibody immune complex formation from chronic infection may activate the classical pathway of complement and cause deposition of immunoglobulins and complement factors in the mesangium and the capillary walls, leading to renal damage [33]. Low levels of plasma complement 3 (C3) and 4 (C4) are frequent findings in HCV-positive patients with cryoglobulinemia, but much less so without it [34–36]. In our study, the activities of the classical and lectin pathways were decreased. Subsequently, activity products of the classical pathway as well as the alternative pathway increased, which is in line with previous theories of HCV-induced complement activation. It also seemed that the urine manifestations correlated with complement activation, possibly indicating that complement activation also contributes to HCV-induced renal manifestations even in their early phase.

There are notable strengths in this study. This is the largest prospective trial comparing the prevalence of early kidney manifestations between HCV positive patients and matched controls. The study population is well characterized with a large number of covariates collected to identify possible biomarkers, which could be applicable in routine clinical practice by gastroenterologists, infectious diseases specialists and addiction medicine specialists who currently treat HCV patients. The robust set of covariates allowed us to adjust for potential confounders. As a limitation of this study, The healthy control group was collected by an occupational health service provider, consisting of healthy individuals participating in their routine health check-ups reimbursed by their employer. For the aforementioned reason and with the proposed exclusion criteria of conditions possibly affecting the kidneys, the median age of the control group was lower. However, the clinical characteristics of the patient and control groups were somewhat similar.

## Conclusions

In conclusion, our study provides real-life, prospective, observational data of early renal findings in a large representative cohort of HCV-positive patients, showing that renal derangements are mostly asymptomatic, not identified using simple dipstick screening tests, and tubular proteinuria is encountered surprisingly often. No patient had MPGN or cryoglobulinemia despite long exposure to an HCV infection. There is paucity of similar prospective data on HCV-positive patients, especially with a similar genotype profile. HCV seems to influence both renal tubular and glomerular cells as judged by different proteinuria profiles and hematuria findings. Tubular cell damage may be the earliest sign of renal dysfunction caused by HCV and could potentially be used to identify patients with a higher risk for developing more severe renal manifestations. Routine urine analyses could be implemented to identify high risk HCV patients for early treatment.

## Supporting information

S1 Fig Proportion of individual kidney manifestations in the HCV+ group with kidney findings. (DOCX)

S1 Table Baseline laboratory values in HCV-positive patients with renal manifestation vs. no renal manifestation. Reference values and units are given where appropriate. Values are expressed as means (range in parenthesis).
(DOCX)

S2 Table Clinical findings in patients with renal manifestation (reported according to patient number). (DOCX)
