# Supplementary figures and images for: Tubular cell damage may be the earliest sign of renal extrahepatic manifestation caused by Hepatitis C
Source: PLoS One. 2021 May 7;16(5):e0251392. doi: 10.1371/journal.pone.0251392 (PMC8104418; doi:10.1371/journal.pone.0251392)

**S1 Fig. Proportion of individual kidney manifestations in the HCV+ group with kidney findings**


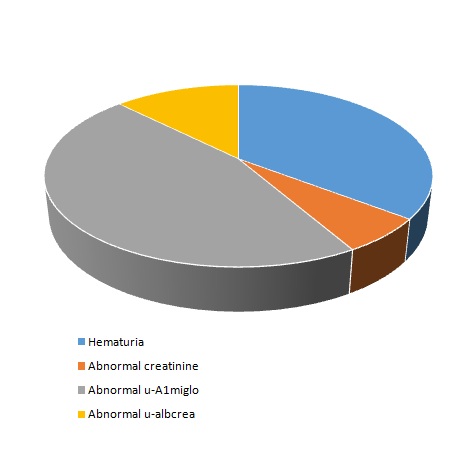

Supplement: S1 Fig — (DOCX) [file pone.0251392.s001.docx]
