# Supplementary material for: Tubular cell damage may be the earliest sign of renal extrahepatic manifestation caused by Hepatitis C
Source: PLoS One. 2021 May 7;16(5):e0251392. doi: 10.1371/journal.pone.0251392 (PMC8104418; doi:10.1371/journal.pone.0251392)
Supplement: S1 Table — Reference values and units are given where appropriate. Values are expressed as means (range in parenthesis). (DOCX) [file pone.0251392.s002.docx]

**S1 Table. Baseline laboratory values in HCV-positive patients with renal manifestation vs. no renal manifestation. Reference values and units are given where appropriate. Values are expressed as means (range in parenthesis).**

| **Baseline variable**  **(reference values, units)** | **Sex** | **HCV positive**  **with renal manifestation**  **n=41** | **HCV positive with no renal manifestation**  **n=169** | **p-value** |
| --- | --- | --- | --- | --- |
| ALT (< 50 M, < 35 F, U/l) | M | 149.1 (41-389) | 135.2 (17-468) | 0.42 |
|  | F | 156.1 (32-909) | 113.5 (8-958) | 0.08 |
| AST (15-45 M, 15-35 F, U/l) | M | 82.7 (33-173) | 84.4 (19-386) | 0.63 |
|  | F | 105.1 (30-676) | 76.4 (22-383) | 0.10 |
| GGT (<60 M, <40 F, U/l) | M | 121.7 (24-367) | 90.3 (11-1168) | 0.06 |
|  | F | 84.1 (12-278) | 50.7 (10-202) | 0.05 |
| Alb (36-45 g/l) | All | 37.8 (20-47) | 38.4 (25-52) | 0.41 |
| Hb (134-167 M, 117-155 F, g/l) | M | 155.1 (123-184) | 151.0 (122-175) | 0.19 |
|  | F | 141.9 (119-155) | 135.8 (115-161) | 0.009 |
| Platelets (150-360 E9/l) | All | 214.1 (93- 385) | 219.3 (54-491) | 0.70 |
| WBC (3.4-8.2 E9/l) | All | 6.7 (4.0-11.6) | 6.6 (3.3-11.6) | 0.66 |
| CRP (< 3 mg/l) | All | 5.0 (3-21) | 3.8 (3-41) | 0.10 |
| *ESR (mm/h) | All | 14.8 (1-91) | 9.5 (1-55) | 0.14 |
| K (3.3-4.9 mmol/l) | All | 3.9 (2.5-4.8) | 3.9 (3.1-4.8) | 0.85 |
| Na (137-143 mmol/l) | All | 138.6 (132 -144) | 138.0 (128-143) | 0.18 |
| Cholesterol (< 5 mmol/l) | All | 4.1 (1.5-6.3) | 4.3 (2.1-10.1) | 0.42 |
| HDL (> 1 mmol/l) | All | 1.3 (0.6-2.8) | 1.3 (0.5-3.4) | 0.61 |
| LDL (< 3 mmol/l) | All | 2.4 (0.5-4.0) | 2.5 (0.9-7.6) | 0.44 |
| Triglycerides (< 1.7 mmol/l) | All | 1.3 (0.3-5.0) | 1.3 (0.3-8.9) | 0.63 |
| HbA1c (20-42 mmol/mol) | All | 37.3 (21-128) | 33.6 (25-82) | 0.55 |

*reference values are age- and sex-dependent: 1-45, M = male, F = female, ALT = alanine aminotransferase, AST = aspartate aminotransferase, GGT = γ glutamyl transpeptidase, alb = albumin, hb = hemoglobin, WBC = white blood cell count, CRP = C-reactive protein, ESR = erythrocyte sedimentation rate, K = potassium, Na = sodium, HDL = high-density lipoprotein, LDL = low-density lipoprotein, HbA1c = glycated hemoglobin
