# Supplementary material for: Tubular cell damage may be the earliest sign of renal extrahepatic manifestation caused by Hepatitis C
Source: PLoS One. 2021 May 7;16(5):e0251392. doi: 10.1371/journal.pone.0251392 (PMC8104418; doi:10.1371/journal.pone.0251392)
Supplement: S2 Table — (DOCX) [file pone.0251392.s003.docx]

**S2 Table. Clinical findings in patients with renal manifestation (reported according to patient number)**

| **Variable** | **Mean (range)**  **n (%)** | **1** | **5** | **8** | **10** | **38** | **42** | **45** | **48** | **49** | **50** | **66** | **71** | **76** | **85** | **87** | **96** | **113** | **123** | **126** | **129** | **130** | **133** | **135** | **146** | **148** | **149** | **158** | **162** | **163** | **166** | **168** | **179** | **181** | **188** | **190** | **193** | **198** | **199** | **206** | **219** | **221** |
| --- | --- | --- | --- | --- | --- | --- | --- | --- | --- | --- | --- | --- | --- | --- | --- | --- | --- | --- | --- | --- | --- | --- | --- | --- | --- | --- | --- | --- | --- | --- | --- | --- | --- | --- | --- | --- | --- | --- | --- | --- | --- | --- |
| **Abnormal hematuria** | 17 (41) |  |  |  | x |  | x |  |  |  |  | x | x | x | x |  |  |  |  |  | x |  | x | x | x |  | x |  |  |  |  | x |  | x |  |  | x | x |  |  | x | x |
| **Abnormal creatinine** | 3 (7) |  |  |  |  |  |  |  |  |  |  |  |  |  |  |  |  |  | x |  |  |  |  |  |  |  |  |  | x |  |  |  |  |  |  | x |  |  |  |  |  |  |
| **Abnormal s-cystatin-C** | 8 (19.5) | x | x |  |  | x |  | x |  |  |  |  |  | x |  |  |  |  |  | x |  |  |  |  |  |  |  |  |  |  |  |  | x |  |  |  | x |  |  |  |  |  |
| **Abnormal U-A1miglo** | 22 (56) | x | x | x |  | x |  | x | x | x | x |  |  |  |  | x | x | x |  | x |  | x |  |  |  | x |  | x |  | x | x | x | x |  | x |  |  |  | x | x |  |  |
| **Abnormal U-AlbCkrea** | 6 (15) |  |  | x |  |  |  |  |  |  |  |  | x | x |  |  |  |  |  | x |  |  | x |  |  |  |  |  |  |  |  |  | x |  |  |  |  |  |  |  |  |  |
| **Genotype** |  | 1 | 3 | 3 | 3 | 1 | 4 | 3 | 3 | 1 | 1 | 3 | 1 | 1 | 3 | 3 | 3 | 3 | 3 | 3 | 1 | 3 | 3 | 1 | 1 | 1 | 3 | 3 | 1 | 1 | 3 | 1 | 3 | 1 | 3 | 1 | 2 | 3 | 1 | 3 | 1 | 1 |
| **Fibrosis stage*** |  | 0 | 0 | 4 | 0 | 2 | 4 | 4 | 0 | 0 | 2 | 0 | 3 | 4 | 0 | 0 | 0 | 0 | 0 | 2 | **-** | 0 | 4 | 0 | 0 | 0 | 4 | 4 | 0 | 2 | 0 | 0 | 0 | 0 | 2 | 0 | 4 | 3 | 3 | 0 | 0 | 0 |
| **Complement analyses** |  |  |  |  |  |  |  |  |  |  |  |  |  |  |  |  |  |  |  |  |  |  |  |  |  |  |  |  |  |  |  |  |  |  |  |  |  |  |  |  |  |  |
| s-C4 ( ref. 0.12-0.42 g/l) | 0.21  (0.04-0.76) |  |  |  |  |  |  |  |  |  |  |  |  |  |  |  |  |  |  |  |  |  |  |  |  |  |  |  |  |  |  |  |  |  |  |  |  |  |  |  |  |  |
| Less than normal n (%) | 4 (11) |  |  | x |  |  |  |  |  |  |  |  |  |  |  |  |  |  |  |  |  |  | x |  |  |  |  |  |  |  |  |  |  |  |  |  | x |  |  | x |  |  |
| s-CH100Cl (ref. > 74 %) | 101.27  (0-200) |  |  |  |  |  |  |  |  |  |  |  |  |  |  |  |  |  |  |  |  |  |  |  |  |  |  |  |  |  |  |  |  |  |  |  |  |  |  |  |  |  |
| Less than normal n (%) | 7 (19) |  |  |  |  |  |  | x |  |  |  |  |  |  |  | x |  |  | x |  | x |  |  |  |  |  |  |  |  |  |  |  |  |  | x |  | x |  |  | x |  |  |
| s-CH100L (ref. > 10 %) | 73.7 (0-198) |  |  |  |  |  |  |  |  |  |  |  |  |  |  |  |  |  |  |  |  |  |  |  |  |  |  |  |  |  |  |  |  |  |  |  |  |  |  |  |  |  |
| Less than normal n (%) | 7 (19) | x |  |  |  |  |  | x | x | x |  |  |  |  |  |  |  |  |  |  |  | x |  |  |  |  |  | x |  |  |  |  |  |  |  |  |  |  |  | x |  |  |
| p-C4d (ref. < 7 µg/ml) | 2.64 (0.5-8.7) |  |  |  |  |  |  |  |  |  |  |  |  |  |  |  |  |  |  |  |  |  |  |  |  |  |  |  |  |  |  |  |  |  |  |  |  |  |  |  |  |  |
| More than normal n (%) | 3 (8) |  |  |  | x |  |  |  |  |  |  |  |  |  |  |  |  |  |  |  |  |  |  |  |  |  |  |  | x |  |  |  |  | x |  |  |  |  |  |  |  |  |
| p-C3d (ref. < 7 U/ml) | 5.52  (2.9-10.7) |  |  |  |  |  |  |  |  |  |  |  |  |  |  |  |  |  |  |  |  |  |  |  |  |  |  |  |  |  |  |  |  |  |  |  |  |  |  |  |  |  |
| More than normal n (%) | 5 (14) |  |  | x |  |  |  |  |  |  | x |  | x |  |  |  |  |  |  | x |  | x |  |  |  |  |  |  |  |  |  |  |  |  |  |  |  |  |  |  |  |  |
| C3nef (pos) n(%) | 4/37 |  |  |  |  |  |  |  |  |  |  |  |  |  |  |  |  |  |  |  | x |  |  |  | x |  |  | x |  |  |  |  |  |  | x |  |  |  |  |  |  |  |
| **Paraproteins** |  |  |  |  |  |  |  |  |  |  |  |  |  |  |  |  |  |  |  |  |  |  |  |  |  |  |  |  |  |  |  |  |  |  |  |  |  |  |  |  |  |  |
| s-IgLcK-V ( ref.6.9-25.6 mg/l) | 28.1  (9.6-104.0) |  |  |  |  |  |  |  |  |  |  |  |  |  |  |  |  |  |  |  |  |  |  |  |  |  |  |  |  |  |  |  |  |  |  |  |  |  |  |  |  |  |
| Outside reference range n (%) | 13 (36) | x | x | x |  |  | x | x |  |  | x |  |  |  |  | x |  |  | x |  |  |  | x |  |  |  |  |  |  | x | x |  | x |  |  |  | x |  |  |  |  |  |
| s-IgLcL-V (ref. 8.6-26.5 mg/l) | 22.4  (9.0-51.9) |  |  |  |  |  |  |  |  |  |  |  |  |  |  |  |  |  |  |  |  |  |  |  |  |  |  |  |  |  |  |  |  |  |  |  |  |  |  |  |  |  |
| Outside reference range n (%) | 9 (25) | x | x | x |  |  |  |  |  |  |  |  | x |  |  |  |  |  |  | x |  |  | x |  |  |  |  |  |  | x |  |  | x |  |  |  | x |  |  |  |  |  |
| s-K/L-s-v-ratio (ref. 0.52 - 1.40) | 1.23  (0.40-2.18) |  |  |  |  |  |  |  |  |  |  |  |  |  |  |  |  |  |  |  |  |  |  |  |  |  |  |  |  |  |  |  |  |  |  |  |  |  |  |  |  |  |
| Outside reference range n (%) | 11 (31) |  | x |  |  |  |  | x |  |  | x | x |  |  |  | x |  |  |  | x | x |  |  |  |  |  |  | x |  | x |  |  | x |  |  |  | x |  |  |  |  |  |
| Serum IgM kappa | 1 (3) |  | x |  |  |  |  |  |  |  |  |  |  |  |  |  |  |  |  |  |  |  |  |  |  |  |  |  |  |  |  |  |  |  |  |  |  |  |  |  |  |  |
| Urine kappa light chain | 2 (7) |  |  |  |  |  |  |  |  |  |  |  |  |  |  |  |  |  |  |  |  |  | x |  |  |  |  |  |  |  |  |  |  |  |  | x |  |  |  |  |  |  |
| Diabetes mellitus | 3/41 (7.3) |  |  | x |  |  |  | x |  |  |  |  |  |  |  |  |  |  |  |  |  |  |  |  |  |  |  |  |  |  |  |  |  |  |  |  | x |  |  |  |  |  |
| Hypertension | 8/41 (19.5) |  | x |  |  | x |  | x |  |  |  |  | x |  |  |  |  |  |  |  |  |  |  |  |  | x |  |  |  |  |  |  | x |  |  |  | x | x |  |  |  |  |

*) Fibrosis stage 0=F0-F1, 2=F2, 3=F3, 4=F4
